# Development and validation of a clinical model for predicting 90-day outcomes after endovascular therapy with adjunctive tirofiban in acute ischemic stroke

**DOI:** 10.3389/fneur.2025.1729880

**Published:** 2026-01-05

**Authors:** Minghui Du, Hanye Yuan, Tianhao Zhang, Zhuqing Luan, Hongchun Wei, Zhongwen Sun, Denglu Liu, Zhigang Liang

**Affiliations:** 1 Department of Neurology, Yuhuangding Hospital Affiliated to Qingdao University, Yantai, China; 2 The Second Clinical Medical College, Binzhou Medical University, Yantai, China; 3 Shandong Provincial Key Laboratory of Neuroimmune Interaction and Regulation, Yantai, China; 4 National Clinical Medical Research Center for Neurological Diseases Regional Subcenter, Yantai, China

**Keywords:** acute ischemic stroke due to large vessel occlusion, endovascular therapy, prediction model, thrombectomy, tirofiban

## Abstract

**Background:**

Endovascular therapy (EVT) represents a cornerstone in the treatment of acute ischemic stroke due to large vessel occlusion (AIS-LVO). Despite high recanalization rates, ineffective microcirculatory reperfusion and early reocclusion can compromise clinical outcomes. The adjunctive use of tirofiban, a glycoprotein IIb/IIIa inhibitor, has been proposed to mitigate these risks, yet identification of patients who may benefit is uncertain. We aimed to develop and validate a clinical prediction model for 90-day poor functional outcome in AIS-LVO patients undergoing EVT with tirofiban.

**Methods:**

We conducted a retrospective cohort study of 177 consecutive AIS-LVO patients who received EVT plus tirofiban at a single academic center. The primary outcome was a poor functional outcome, defined as modified Rankin Scale score 3–6 at 90 days. Secondary outcomes included successful reperfusion (mTICI 2b–3), symptomatic intracranial hemorrhage (sICH), and 90-day mortality. Using 70% of the cohort for model development, we constructed predictors via multivariable logistic regression and machine learning approaches (including XGBoost, Random Forest, and others). Predictors comprised baseline clinical, imaging, and procedural variables. Model performance was assessed by area under the curve (AUC), calibration plots, and decision curve analysis (DCA), sensitivity, specificity, precision.

**Results:**

Poor functional outcome was observed in 50.8% of patients. Multivariable analysis identified stroke-associated pneumonia (OR 7.56, 95% CI 2.75–20.77), higher baseline NIHSS score (OR 1.13, 95% CI 1.03–1.24), and smoking history (OR 2.86, 95% CI 1.19–6.85) as independent predictors of poor outcome, while successful reperfusion was protective (OR 0.06, 95% CI 0.01–0.57). The final nomogram model demonstrated good discrimination (AUC 0.83, 95% CI 0.75–0.90) and calibration (Hosmer–Lemeshow test, *p* = 0.539).

**Conclusion:**

We developed and validated a pragmatic prediction model incorporating readily available clinical and procedural variables to estimate the risk of 90-day poor outcome in AIS-LVO patients treated with EVT and tirofiban. This tool may assist clinicians in individualized outcome prediction and inform adjunctive antithrombotic strategies in neurovascular care.

## 1 Introduction

Acute ischaemic stroke (AIS) remains a leading cause of disability and mortality worldwide, posing substantial challenges to public health systems and imposing considerable socioeconomic burdens on patients, families, and communities (1). Large vessel occlusion (LVO) is responsible for more than 30% of AIS cases and is associated with particularly severe neurological deficits and poor prognosis (2). Endovascular therapy (EVT) has markedly improved functional outcomes in eligible patients compared with medical management alone, establishing itself as a standard of care for AIS-LVO (3, 4). Nevertheless, despite high rates of successful recanalization [modified thrombolysis in cerebral infarction (mTICI) score 2b–3], 40–50% of patients still experience poor functional outcomes [modified Rankin Scale (mRS) score 3–6] at 90 days (5), highlighting the need for adjunctive strategies to optimize EVT benefits.

Platelet activation and aggregation play a central role in the development of in-situ thrombosis and early re-occlusion following EVT. Tirofiban, a highly selective, short-acting glycoprotein IIb/IIIa inhibitor, antagonizes the final common pathway of platelet aggregation. Growing evidence from clinical studies and meta-analyses suggests that intravenous tirofiban administered during EVT may improve reperfusion outcomes, reduce re-occlusion rates, and potentially enhance functional recovery, especially in select populations or as rescue therapy (6–8). However, uncertainty persists regarding its safety profile, particularly concerning symptomatic intracranial hemorrhage (sICH), which has restricted its routine adoption. Current use remains largely empiric, guided by institutional protocols rather than evidence-based, individualized prognostic tools.

For AIS patients undergoing EVT, intravenous administration of tirofiban demonstrates favorable efficacy and safety profiles, promoting functional recovery and reducing mortality (9–11). Accurate outcome prediction for AIS patients undergoing EVT with adjunctive tirofiban is essential for personalized treatment decisions, patient counseling, and future trial design. Although several prognostic models exist for general EVT populations (e.g., THRIVE, HIAT, SPAN-100) (12–14), none account for the specific effect of tirofiban. There is a compelling need for tailored predictive instruments that incorporate detailed treatment parameters. Therefore, this study aimed to develop and validate a prognostic model specifically designed to predict 90-day poor functional outcome in AIS-LVO patients treated with EVT plus intravenous tirofiban.

## 2 Materials and methods

### 2.1 Participants

We conducted a retrospective cohort study of consecutive AIS-LVO patients treated at Yantai Yuhuangding Hospital Affiliated to Qingdao University between January 2022 and May 2025. Inclusion criteria were: (1) age ≥18 years; (2) admission NIHSS score 0–20 (inclusive), pre-stroke mRS score ≤1; (3) AIS with intracranial large vessel occlusion confirmed by cranial computed tomography (CT) or magnetic resonance imaging (MRI); (4) EVT initiation within 24 h of symptom onset (or last known well); and (5) administration of intravenous tirofiban during or peri-procedurally with EVT. Exclusion criteria included: (1) intracranial hemorrhage confirmed by cranial CT or MRI; (2) wake-up stroke or unclear onset time; (3) discharge or death within 24 h post-surgery; and (4) contraindications to tirofiban (e.g., active bleeding, known hypersensitivity to tirofiban, severe thrombocytopenia, recent major surgery/trauma, or uncontrolled severe hypertension). Tirofiban was administered as an intra-arterial or intravenous bolus of 0.5–0.6 mg at a rate of 0.05 mg/min during the procedure, followed by continuous intravenous infusion at 0.1 μg/kg·min after the procedure. The study was approved by the Ethics Committee of Yantai Yuhuangding Hospital Affiliated to Qingdao University. Informed consent was waived due to the retrospective design and use of anonymized data, in compliance with local ethical regulations.

### 2.2 Data collection

Data were extracted from electronic medical records and dedicated stroke databases by trained research personnel. Data collected included baseline demographics, laboratory results, surgical details, and primary and secondary outcomes. Baseline data comprised sex, age, admission blood glucose (BG), pre-stroke mRS and admission NIHSS scores, Alberta Stroke Program Early CT Score (ASPECTS), stroke-associated pneumonia (SAP), thrombolytic therapy (bridging), stroke etiology [Trial of Org 10172 in Acute Stroke Treatment (TOAST) classification], comorbidities (including hypertension, diabetes mellitus, atrial fibrillation, hyperlipidemia, history of stroke or transient ischemic attack, and coronary artery disease), as well as alcoholism and smoking history. Laboratory data included complete blood count, biochemistry, coagulation profile, and electrolytes. Surgical data comprised onset-to-puncture (OTP) time, anesthesia type, number of mechanical thrombectomy (MT) attempts (stent retriever or catheter suction), procedure duration, occlusion site, combined interventions (balloon angioplasty or stenting), and post-procedural mTICI grade.

Primary outcome: A professionally trained neurologist assessed patients via telephone or outpatient follow-up 90 days after enrollment and calculated the mRS score. A 90-day mRS score of 0–2 was defined as a good functional outcome (functional independence), while a score of 3–6 was defined as a poor functional outcome (functional dependence or death). Secondary outcomes included: (1) Successful reperfusion (postoperative mTICI grade 2b–3); (2) sICH, defined according to the Heidelberg Bleeding Classification within 36 h (parenchymal hematoma type 1 or 2, subarachnoid hemorrhage, or intraventricular hemorrhage associated with an increase of ≥4 points in NIHSS score from baseline or leading to death); (3) Any intracranial hemorrhage (ICH) detected on follow-up imaging; and (4) 90-day mortality, defined as an mRS score of 6.

### 2.3 Statistical analysis

The cohort was randomly divided into a training set (70%) and a validation set (30%). Model development adhered to the transparent reporting of a multivariable prediction model for individual prognosis or diagnosis (TRIPOD) guidelines. All statistical analyses were performed using R software (version 4.4.0). Candidate predictors were selected based on clinical relevance and existing literature. Missing rates of all variables were <3% (range: 0.5–2.8%), and cases with missing key variables (SAP, NIHSS, mTICI) were excluded (*n* = 6, 3.4%).

#### 2.3.1 Feature selection and model development

The normality of continuous variables was assessed using the Kolmogorov–Smirnov test. Predictors associated with poor outcomes in AIS-LVO patients were initially identified via least absolute shrinkage and selection operator (LASSO) regression analysis with ten-fold cross-validation, using the one standard error (1se) rule to select the most parsimonious model. Variables selected by LASSO were then incorporated into multivariable logistic regression analysis to build the predictive model. A nomogram was constructed based on the final model’s predictors. Hyperparameter tuning for each ML algorithm was conducted using a 5-fold cross-validated grid search within the training set to optimize model performance and mitigate overfitting. The best-performing set of hyperparameters for each model was selected based on the highest cross-validated AUC.

#### 2.3.2 Model validation and performance evaluation

Internal validation of the logistic regression model was performed using bootstrap resampling (1,000 iterations) to correct for optimism. The final model’s performance was further evaluated in the independent validation set. For both the logistic and ML models, discrimination was evaluated using the area under the receiver operating characteristic curve (AUC). Calibration plots and the Hosmer–Lemeshow test assessed the agreement between predicted and observed outcomes. Decision curve analysis (DCA) was applied to assess the net clinical benefit across a range of threshold probabilities.

## 3 Results

### 3.1 General clinical characteristics

**Figure 1:**
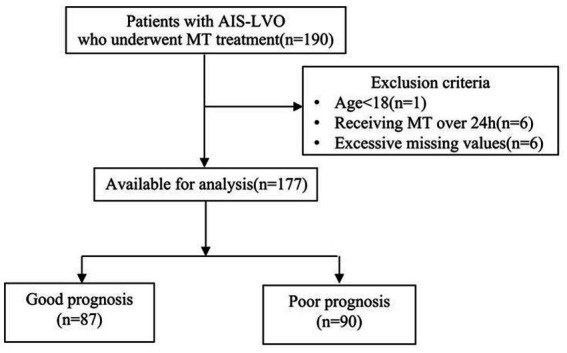
Flow chart of patient inclusion.

The patient selection process is outlined in Figure 1. From the initial cohort, 177 AIS-LVO patients who underwent EVT with adjunctive tirofiban were included in the final analysis. Of these, 87 patients (49.2%) achieved a good functional outcome (mRS 0–2), while 90 patients (50.8%) had a poor functional outcome (mRS 3–6).

### 3.2 Baseline and laboratory data between two groups

Table 1 summarizes the baseline and clinical characteristics of all enrolled patients. The good outcome group comprised 67 men and 20 women, compared to 63 men and 27 women in the poor outcome group. Regarding treatment outcomes, successful revascularization was achieved in 85 patients in the good outcome group, with one case of sICH. In the poor outcome group, 74 patients attained successful revascularization, while six developed sICH. In our cohort, the overall sICH rate was 4.0% (7/177), which is consistent with the 3.8% rate reported in the RESCUE BT trial (a key study on tirofiban in AIS-LVO), confirming the representativeness of our study population. Significant differences were observed between the groups for the following variables: smoking history, baseline NIHSS, stroke-associated pneumonia, WBC, admission blood glucose, serum magnesium level, number of MT attempts, onset-to-puncture time <6 h, mTICI 2b–3, sICH, and any ICH.

**Table 1:** Baseline characteristics of the study population stratified by 90-day functional outcome.

Variable	Good prognosis (mRS 0–2) (*n* = 87)	Poor prognosis (mRS 3–6) (*n* = 90)	*p*-value
Demographic characteristics			
Age, years	67 (57.50, 71.00)	67.5 (59.25, 72.75)	0.155
Male, *n* (%)	67 (77.01)	63 (70.00)	0.376
BMI (kg/m^2^)	25.80 (24.00, 27.70)	26.00 (24.25, 28.00)	0.480
Vascular risk factors, *n* (%)			
Hypertension	50 (57.47)	53 (58.89)	0.969
Diabetes mellitus	21 (24.14)	32 (35.56)	0.135
Coronary heart disease	33 (37.93)	36 (40.00)	0.898
Atrial fibrillation	25 (28.74)	27 (30.00)	0.984
Stroke or tia	25 (28.74)	25 (27.78)	1.000
Cancer	8 (9.20)	9 (10.00)	1.000
Smoking history	28 (32.18)	49 (54.44)	**0.005**
Alcoholism	33 (37.93)	38 (42.22)	0.668
Clinical data			
Baseline NIHSS, score	10.00 (6.00, 14.00)	14.00 (11.00, 16.75)	**<0.001**
Baseline ASPECTS, score	8.00 (7.00, 9.00)	8.00 (6.00, 9.00)	0.184
Bridging therapy (*n*, %)	40 (45.98)	42 (46.67)	1.000
Stroke-associated pneumonia (*n*, %)	44 (50.57)	80 (88.89)	**<0.001**
Preoperative MRI (*n*, %)	42 (48.28)	50 (55.56)	0.413
Laboratory data			
CRP (mg/L)	3.44 (1.23, 13.50)	3.33 (1.10, 11.98)	0.613
BNP (pg/L)	44.82 (20.55, 165.19)	66.86 (20.75, 190.53)	0.561
RBC (×10^12^/L)	4.86 (4.50, 5.14)	4.80 (4.41, 5.12)	0.286
WBC (×10^9^/L)	8.30 (6.64, 9.93)	9.75 (7.26, 11.81)	**0.007**
PLT (×10^9^/L)	204.00 (181.25, 257.25)	214.50 (173.25, 250.50)	0.856
Hb (g/L)	147.00 (137.50, 155.00)	144.50 (134.00, 153.00)	0.329
HbA1c (%)	6.00 (5.60, 6.90)	6.05 (5.80, 7.68)	0.196
GLU (mmol/L)	7.10 (5.90, 8.86)	8.21 (6.41, 10.04)	**0.014**
AST (μ/L)	19.00 (16.00, 24.00)	19.00 (17.00, 26.00)	0.380
ALT (μ/L)	19.00 (14.00, 24.50)	16.30 (12.25, 24.00)	0.362
Urea (mmol/L)	4.87 (3.98, 5.77)	4.88 (3.91, 6.32)	0.541
Cr (μmol/L)	61.00 (52.00, 72.00)	62.00 (52.25, 76.00)	0.661
UA (μmol/L)	309.00 (259.50, 369.00)	288.00 (238.00, 348.00)	0.102
ALB (g/L)	36.70 ± 3.83	35.98 ± 4.18	0.232
DBIL (μmol/L)	2.70 (2.30, 3.65)	2.80 (2.20, 3.40)	0.424
IBIL (μmol/L)	12.20 (10.05, 17.30)	12.60 (9.30, 15.60)	0.311
TC (mmol/L)	4.49 (3.75, 5.34)	4.43 (3.90, 5.09)	0.738
TG (mmol/L)	1.09 (0.78, 1.46)	0.99 (0.78, 1.45)	0.564
HDL-C (mmol/L)	1.13 (0.96, 1.23)	1.18 (1.02, 1.35)	0.060
LDL-C (mmol/L)	2.76 (2.22, 3.50)	2.63 (2.10, 3.35)	0.527
Lipoproteins (mg/L)	180.00 (75.50, 301.50)	113.00 (61.00, 299.00)	0.402
HCY (μmol/L)	12.20 (9.90, 14.85)	10.50 (8.50, 12.70)	0.060
PT (sec)	11.80 (11.30, 12.50)	12.10 (11.50, 13.00)	0.294
APTT (sec)	29.60 (27.10, 32.80)	29.30 (26.60, 31.30)	0.288
TT (sec)	17.00 (16.15, 17.70)	16.90 (16.10, 17.70)	0.947
INR	1.00 (0.97, 1.06)	1.02 (0.97, 1.08)	0.478
FIB (g/L)	3.06 (2.53, 3.64)	3.20 (2.56, 3.67)	0.328
D-Dimer (mg/L)	0.85 (0.63, 1.70)	1.11 (0.73, 2.83)	0.070
Na (mmol/L)	139.80 (138.10, 141.50)	140.05 (138.20, 141.90)	0.289
Cl (mmol/L)	106.60 (103.55, 108.70)	106.20 (104.30, 108.45)	0.825
Ca (mmol/L)	2.15 (2.07, 2.24)	2.12 (2.04, 2.22)	0.394
K (mmol/L)	3.96 (3.78, 4.16)	3.94 (3.74, 4.09)	0.414
Mg (mmol/L)	0.88 (0.83, 0.95)	0.85 (0.79, 0.91)	**0.017**
Infarction site (*n*, %)			0.286
Anterior circulation	68 (78.16)	63 (70.00)	
Posterior circulation	19 (21.84)	27 (30.00)	
TOAST classification, *n* (%)			0.819
Large artery atherosclerosis	59 (67.82)	57 (63.33)	
Cardioembolism	26 (29.89)	31 (34.44)	
Others	2 (2.30)	2 (2.22)	
Surgical data			
Time from puncture to recanalization, min	105 (80, 135)	120 (85, 150)	0.098
Number of MT (suction/stent)	1.00 (1.00, 2.00)	2.00 (1.00, 3.00)	**0.010**
Onset-to-puncture time <6 h	58 (66.67)	41 (45.56)	**0.007**
General anesthesia	21 (24.14)	29 (32.22)	0.304
Other operations (*n*, %)			0.079
Balloon dilatation	9 (10.34)	10 (11.1)	
Stent implantation	1 (1.15)	7 (7.78)	
Balloon + stent	32 (36.78)	22 (24.44)	
mTICI 2b–3 (*n*, %)	85 (97.70)	74 (82.22)	**0.002**
sICH (*n*, %)	1 (1.15)	6 (6.67)	**0.018**
ICH (*n*, %)	12 (13.79)	31 (34.44)	**0.002**

CRP, C-reactive protein; BNP, brain natriuretic peptide; HDL-C, high-density lipoprotein cholesterol; LDL-C, low-density lipoprotein cholesterol; HCY, homocysteine; INR, ‌international normalized ratio; NIHSS, National Institutes of Health Stroke Scale; mTICI, modified thrombolysis in cerebral infarction; sICH, symptomatic intracranial hemorrhage; HbA1c, glycated hemoglobin. The bolded values denote factors exhibiting statistically significant differences between the two screened groups (*p* < 0.05).

### 3.3 LASSO regression analysis and predictor selection

**Figure 2:**
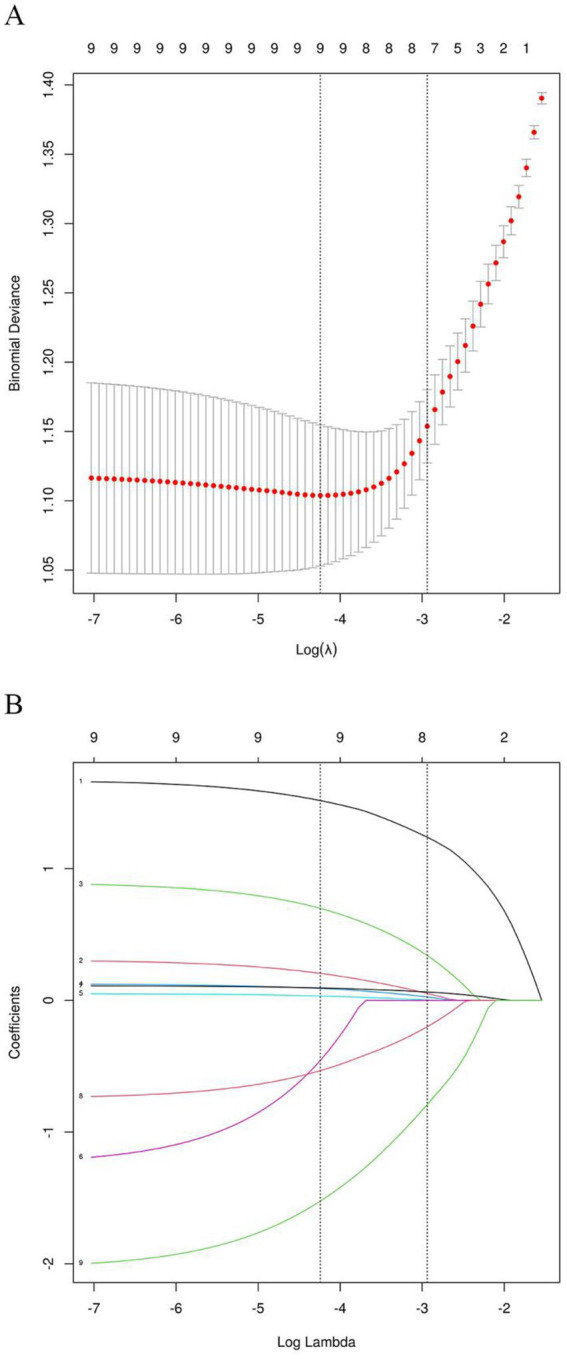
Cross-validation and LASSO regression for predictor selection. **(A)** Cross-validation process. **(B)** LASSO regression analysis.

**Figure 3:**
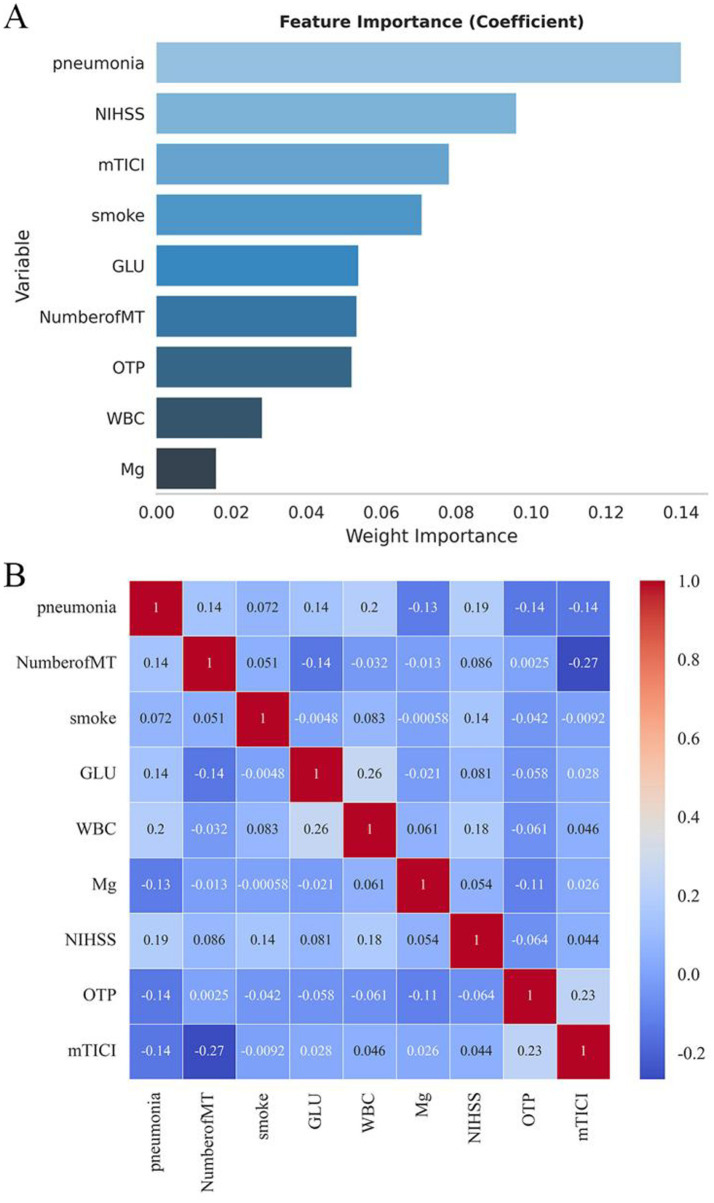
Feature importance ranking and correlation analysis of LASSO-selected variables. **(A)** Feature importance. **(B)** Correlation analysis.

Candidate predictors identified through univariate analysis were further refined using LASSO regression. The optimal regularization parameter (*λ*) was determined via ten-fold cross-validation based on the one standard error (1se) rule (Figure 2A). Applying this criterion (*λ* = 0.053) selected eight predictors: stroke-associated pneumonia, baseline NIHSS score, mTICI 2b–3, smoking history, admission glucose (GLU), number of MT attempts, onset-to-puncture time <6 h, and white blood cell count (WBC). Serum magnesium level was excluded during this process (Figure 2B). LASSO-selected variables underwent feature importance ranking and correlation analysis (Figures 3A,B).

Subsequently, multivariable logistic regression was performed using these variables. The analysis identified four independent predictors of poor functional outcome: stroke-associated pneumonia, baseline NIHSS score, successful reperfusion (mTICI 2b–3), and smoking history. Variables such as GLU, number of MT attempts, onset-to-puncture time <6 h, and WBC, while selected by LASSO, did not retain independent significance (*p* > 0.05) in the multivariable model alongside the four stronger predictors. Specifically, stroke-associated pneumonia (OR 7.56, 95% CI 2.75–20.77), higher baseline NIHSS score (OR 1.13 per point, 95% CI 1.03–1.24), and smoking history (OR 2.86, 95% CI 1.19–6.85) were significantly associated with an increased risk of poor outcome. In contrast, successful reperfusion (mTICI 2b–3; OR 0.06, 95% CI 0.01–0.57) was associated with improved outcomes (Table 2). The final predictive model was based on these four variables.

**Table 2:** Multifactor logistic regression results for predictors of poor prognosis in AIS-LVO patients.

Predictors	*β*	SE	Odds ratio (95% CI)	*p*-value
Pneumonia	2.02	0.52	7.56 (2.75 to 20.77)	<0.001
Smoke	1.05	0.45	2.86 (1.19 to 6.85)	0.018
mTICI	−2.75	1.12	0.06 (0.01 to 0.57)	0.014
NIHSS score	0.12	0.05	1.13 (1.03 to 1.24)	0.008

Pneumonia: 1 = stroke-associated pneumonia, 0 = none; mTICI: 1 = mTICI 2b–3, 0 = mTICI 0–2a; NIHSS: continuous variable (per 1-point increase).

### 3.4 Construction and validation of nomogram for predicting prognosis of patients

**Figure 4:**
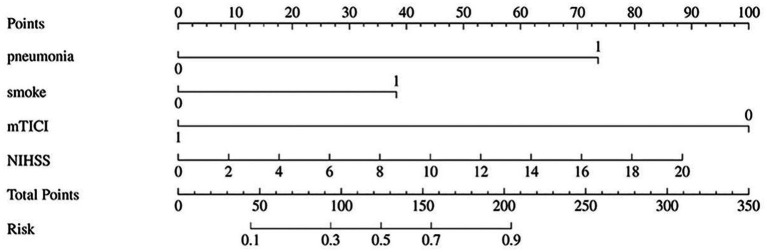
Nomogram for predicting poor prognosis in AIS-LVO patients.

Based on the four independent predictors identified by multivariate logistic regression, a nomogram was developed to estimate individual risk of poor outcome (Figure 4). Each predictor was assigned a score on a scale from 0 to 100. The sum of these scores yields a total point value, corresponding to a probability of a poor outcome. Higher total points indicated increased risk.

**Figure 5:**
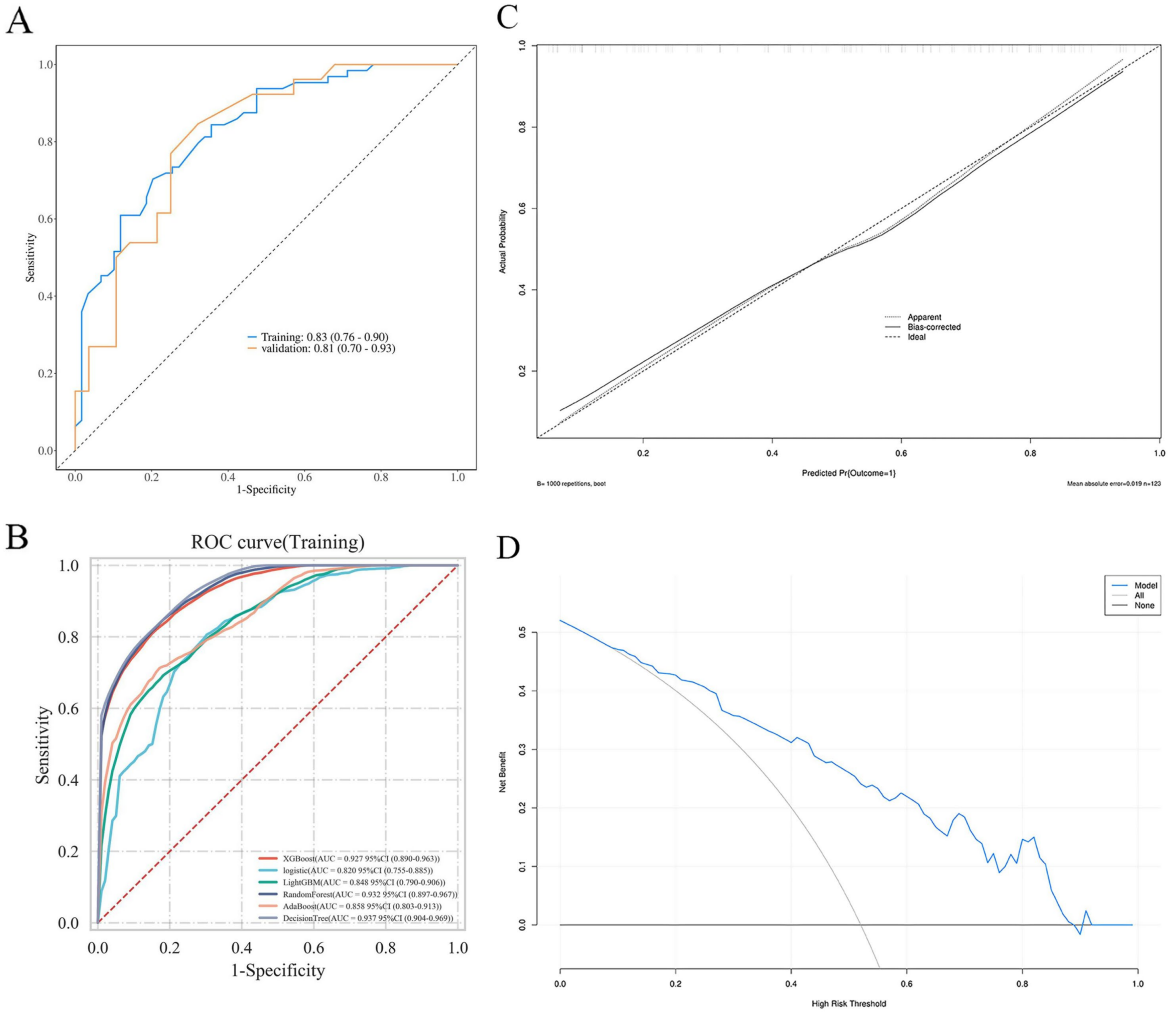
ROC curves **(A,B)**, calibration curves **(C)**, and DCA curve **(D)** of the nomogram.

The predictive performance of the nomogram was evaluated using ROC analysis. The AUC was 0.83 (95% CI: 0.78–0.88) in the training set and 0.81 (95% CI: 0.73–0.89) in the validation set, indicating good discriminative ability (Figure 5A). The model’s performance metrics on the validation set were: Sensitivity 0.75, Specificity 0.77, Precision 0.78, NPV 0.74. For comparison, several machine learning algorithms were applied, including XGBoost, LightGBM, Random Forest, AdaBoost, and Decision Tree. Their respective AUC values were as follows: Decision Tree: 0.937 (95% CI: 0.904–0.969), Random Forest: 0.932 (95% CI: 0.897–0.967), XGBoost: 0.927 (95% CI: 0.890–0.963), LightGBM: 0.848 (95% CI: 0.790–0.906), and AdaBoost: 0.858 (95% CI: 0.803–0.913) (Figure 5B). While tree-based models (Decision Tree, Random Forest, XGBoost) achieved notably higher AUCs on the training data, their performance on the independent validation set was similar to the logistic regression model, suggesting some degree of overfitting to the training data despite hyperparameter tuning. Calibration curves showed good agreement between predicted probabilities and observed outcomes for the logistic model (Hosmer–Lemeshow test, *p* = 0.539) (Figure 5C). DCA indicated favorable clinical utility of the models across a wide range of threshold probabilities (Figure 5D).

## 4 Discussion

This study developed and validated the tirofiban-EVT prognostic (TEP) model, a clinical prediction tool designed to estimate the probability of 90-day poor functional outcomes in AIS-LVO patients treated with EVT with adjunctive tirofiban. Unlike established general prognostic models such as THRIVE, HIAT, and SPAN-100—which do not incorporate the effect of tirofiban—the TEP model integrates treatment-specific variables alongside key clinical and imaging predictors. It thereby addresses an important gap in personalized outcome prediction for this patient population. The model identified stroke-associated pneumonia, baseline NIHSS score, smoking history, and unsuccessful reperfusion (mTICI 0–2a) as independent predictors of poor prognosis. With strong discrimination (AUC: 0.83 in training, 0.81 in validation) and excellent calibration (Hosmer–Lemeshow test, *p* = 0.539), the TEP model demonstrates reliability suitable for clinical application.

### 4.1 Mechanistic and clinical insights into key predictors

The NIHSS score is widely used to evaluate the severity of neurological deficits in acute stroke (15). Higher NIHSS scores are associated with larger infarct volumes and significant cerebral edema, indicating a poorer prognosis (16, 17). Evidence suggests that the risk of unfavorable outcomes after revascularization increases progressively with higher NIHSS scores (18–20). A meta-analysis further demonstrated that EVT provides greater benefit over medical therapy alone in patients with NIHSS scores exceeding 20 (21). Considering that patients with elevated NIHSS scores are at increased risk of poor outcomes after EVT—and considering the limited adoption of tirofiban—our study excluded cases of very severe stroke. Nonetheless, EVT remains capable of facilitating reperfusion of ischemic brain tissue and promoting neurological recovery compared to pharmacologic treatment alone.

Successful reperfusion, defined as mTICI 2b–3, is a critical determinant of favorable outcomes post-EVT in AIS-LVO. Recent evidence suggests that achieving higher reperfusion grades (eTICI 2c–3) confers even better functional outcomes in both anterior and posterior circulation strokes (22–24). Importantly, 17.8% of patients with poor outcomes in our cohort still achieved mTICI 2b–3 reperfusion, emphasizing that technical success does not guarantee clinical success and underscores the need for ongoing vigilance. SAP emerged as the strongest predictor of poor prognosis (OR 7.56), consistent with substantial evidence that post-stroke infections exacerbate secondary brain injury (25, 26). Mechanisms include neutrophil activation, inflammatory mediator release, endothelial dysfunction, axonal damage, and increased blood–brain barrier permeability. This finding highlights the critical importance of implementing rigorous infection prevention strategies in patients receiving EVT with tirofiban. Smoking is a well-established stroke risk factor (27). Furthermore, research by Matsuo et al. (28) indicates that recent quitters among current and former smokers are also at higher risk of functional impairment at 3 months post-stroke. Smoking history was defined as “current or past smoking for ≥1 year” (consistent with the WHO definition), and current smokers accounted for 72.3% (35/48) of the smoking subgroup, suggesting that long-term smoking-related vascular damage may reduce the protective effect of EVT plus tirofiba.

Prolonged procedure time in AIS-LVO is associated with poorer outcomes and higher complication rates (29). Extended intervention duration often reflects greater anatomical complexity, technical challenges during recanalization, and an elevated risk of iatrogenic vascular injury, collectively increasing the likelihood of reocclusion and adverse events (23, 30).

### 4.2 Comparison with existing prognostic models

The TEP model offers distinct advantages over general EVT prognostic models. For instance, while the THRIVE score incorporates age, NIHSS, and ASPECTS, it does not account for treatment-specific variables like tirofiban use or infection-related complications. Similarly, the SPAN-100 index relies solely on age and NIHSS, omitting reperfusion status. In contrast, the TEP model integrates tirofiban-relevant predictors and SAP, providing a more tailored prognostic tool for this specific treatment context. Moreover, the TEP model achieved an AUC of 0.83 for predicting 90-day outcomes, outperforming reported AUCs for THRIVE (0.76) and SPAN-100 (0.78), highlighting its enhanced discriminative ability within the EVT-plus-tirofiban population.

### 4.3 Machine learning

Machine learning is rapidly evolving and shows promise in outperforming traditional scoring systems for predicting outcomes in AIS patients undergoing MT (31, 32). Previous studies employing machine learning for post-MT outcome prediction often utilized a limited range of algorithms, potentially restricting the identification of optimal models (33, 34). Rigorous data preprocessing and feature engineering are crucial, as neglecting dimensionality reduction or highly correlated features can increase model complexity and overfitting risk. In our study, tree-based ensemble models (Decision Tree, Random Forest, XGBoost) achieved high predictive accuracy (AUC >0.9) on the training data. However, these models exhibited a tendency toward overfitting. Consequently, we selected the logistic regression model for its favorable balance of performance and clinical interpretability. Notably, all evaluated models demonstrated predictive value significantly exceeding random chance.

### 4.4 Considerations on NIHSS selection and hemorrhagic risk in cardioembolism

The selection of patients with admission NIHSS scores between 0 and 20 in this study warrants specific discussion regarding safety, particularly in the context of stroke etiology. Cardioembolic strokes are often associated with larger infarct cores and a higher inherent risk of hemorrhagic transformation compared to atherosclerotic strokes, due to factors such as sudden vessel occlusion and potential reperfusion injury. The adjunctive use of an antiplatelet agent like tirofiban could theoretically exacerbate this risk.

Our decision to exclude patients with very severe deficits (NIHSS >20) was based on the established correlation between extreme stroke severity and increased rates of symptomatic intracranial hemorrhage and mortality following endovascular therapy. By focusing on the moderate-to-severe spectrum (NIHSS 0–20), we aimed to study a population where the potential benefit of adjunctive tirofiban might outweigh the procedural risks, including hemorrhage. This selective enrollment likely contributed to the manageable overall sICH rate of 4.0% in our cohort, which aligns with rates reported in pivotal trials like RESCUE BT. Consequently, this safety profile within the defined NIHSS range may have attenuated observable outcome differences between etiologic subgroups (LAA vs. CE) in our study, as the higher-risk cardioembolic patients were inherently those with more severe presentations. Future studies with larger, multicenter datasets are warranted to evaluate the interaction between stroke etiology, baseline NIHSS, and the net clinical benefit of tirofiban, which could further refine personalized prognosis in this population.

### 4.5 Limitations of the present study

This study has several limitations. First, the single-center retrospective design and lack of external validation may introduce selection bias and limit the generalizability of the findings. Although substantial, the sample size remains modest; future expansion is necessary to enhance the nomogram’s robustness. Second, as enrolled patients had admission NIHSS scores between 0 and 20, our results may not apply to patients with very severe deficits (NIHSS >20). Third, several potential outcome modifiers were not accounted for, including the specific thrombolytic agent used (alteplase vs. tenecteplase) and detailed perioperative blood pressure management strategies. Fourth, infusion duration of tirofiban could affect the reproducibility and generalizability of our results. Future studies should establish standardized tirofiban dosing regimens. Therefore, larger multicenter studies with external validation are essential to confirm and refine the predictive accuracy of the TEP model.

## 5 Conclusion

We developed and validated the tirofiban-EVT prognostic (TEP) model, a clinical tool that integrates baseline clinical and imaging features with procedural outcomes to predict the probability of 90-day poor functional outcome in patients with AIS-LVO undergoing EVT with adjunctive tirofiban. The model identified baseline NIHSS score, smoking history, stroke-associated pneumonia, and successful reperfusion (mTICI 2b–3) as key prognostic variables and demonstrated good discrimination and calibration. The TEP model helps address an important gap in personalized prognosis for this treatment strategy and provides a basis for clinical decision-support and future research aimed at optimizing tirofiban use in endovascular stroke care.

## Data Availability

The raw data supporting the conclusions of this article will be made available by the authors, without undue reservation.
